# Differentiation of Feeding Behaviors Based on Masseter and Supra-Hyoid Muscle Activity

**DOI:** 10.3389/fphys.2020.00618

**Published:** 2020-06-12

**Authors:** Fumiko Uehara, Kazuhiro Hori, Kazuhiro Murakami, Jumpei Okawa, Takahiro Ono

**Affiliations:** ^1^Division of Comprehensive Prosthodontics, Faculty of Dentistry & Graduate School of Medical and Dental Sciences, Niigata University, Niigata, Japan; ^2^Department of Prosthodontics, Gerodontology and Oral Rehabilitation, Osaka University Graduate School of Dentistry, Osaka, Japan

**Keywords:** muscle activity, tongue, mastication, squeezing, Lissajous figure

## Abstract

Older adults with disorders of mastication and swallowing are often fed soft foods such as jelly or puree. The texture of such semi-solid foods allows them to be squeezed between the tongue and palate rather than being chewed. However, it is difficult to visually identify such strategies for the oral processing of food. This study aimed to test the hypothesis that there is a difference in the sequential coordination between the masseter and supra-hyoid muscles, and to identify feeding behaviors such as chewing and squeezing using electromyography. Seventeen male subjects (mean age: 30.8 years) were recruited. Four kinds of gels were prepared (two kinds of fracture force and fracture strain) as test samples. Subjects were instructed to consume the gels in three ways: squeezing with the tongue, chewing with the teeth and eating freely until swallowing. The amount of squeezing/chewing and the consumption time was unlimited. The masseter and supra-hyoid muscle activity were recorded during the entire consumption time and videofluorography was simultaneously recorded during each ingestion. Lissajous figures were made from the electromyographic activity of the two groups of muscles during the first stroke, and a regression line was made to determine the gradient of each figure to compare squeezing and chewing using the Mann–Whitney *U*-test. The masseter and supra-hyoid muscles were active simultaneously during squeezing with the tongue. However, the masseter was active after the supra-hyoid during chewing. The gradient of the regression line from the Lissajous figures between the masseter and supra-hyoid muscle activity was positive during squeezing, but negative during chewing. Analysis of the ROC curve showed that the cutoff value of the gradient for differentiating feeding behaviors was 0.097, with a sensitivity of 95.3% and specificity of 98.4%. When we allocated 68 free intakes into squeezing and chewing according to this cutoff value, we could distinguish with good precision, and the accuracy, sensitivity, and specificity were 86.8, 91.1, and 66.7% respectively. These results suggest that certain aspects of muscle activity differed among oral processing methods. Lissajous analysis of muscle activity was useful for identifying ingestion behaviors.

## Introduction

Accidental suffocation is the most common form of accidental death in older adults, and in Japan, half of these deaths are caused by respiratory obstruction resulting from food aspiration (Kiyohara et al., 2018; Cabinet Office Government of Japan, 2019). Therefore, providing meals for older adults that can be consumed safely without suffocation is an urgent issue. Risk factors related to suffocation in older adults can be divided into human factors and factors related to the physical properties of food. The cross-sectional area of the lower end of the oropharyngeal cavity is significantly smaller in older adults than in younger people (Ariyoshi et al., 2013) possibly as a result of changes in the shape of the pharyngeal cavity caused by age-related drooping of the larynx. Another study reported age-related weakness in muscles and deterioration of the swallowing reflex (Nishikubo et al., 2015). Risk factors for suffocation in aged care facility residents are related to cognitive function, the presence of meal independence, and the presence of molar occlusion (Ariyoshi et al., 2013). Swallowing without chewing and food thrusting are often seen in older adults with cognitive impairment even when they are capable of eating meals independently (Samuels and Chadwick, 2006). To prevent suffocation of older adults who need long-term care, it is necessary to assess their masticatory and swallowing function and provide appropriate assistance with meal planning, such as selecting the food type and adjusting the size and texture according to their masticatory ability.

Semi-solid food such as jelly tends to be squeezed between the tongue and palate rather than being chewed (Ishihara et al., 2014). However, it is difficult to identify such strategies for oral processing of food by visual observation. Additionally, self-reporting by older adults with cognitive decline may not match the actual feeding behaviors. Appropriate bolus formation is important for swallowing, and it is necessary to match the meal form with the feeding behavior. Mastication is defined as the process of chewing food for swallowing and digestion (Ferro et al., 2017) or the process of food ingestion, crushing, mixing with saliva, and finally forming a bolus. In the process mode developed by Palmer et al. (1992) the processes associated with mastication consist of food intake, transfer to the molars (stage I transport), food processing, and bolus transport to the oropharynx before swallowing (stage II transport) (Hiiemae and Palmer, 1999). If one of these processes fails, the most important masticatory purpose of forming a swallowable bolus cannot be achieved. There are many kinds of research on muscle activity during mastication, where the main research subjects were the masseter and temporal muscles with solid foods either natural or no edible as test foods (Foster et al., 2006; Iguchi et al., 2015; Révérend et al., 2016; Bilt and Abbink, 2017). However, few studies have investigated mastication of semi-solid foods (Kohyama et al., 2016).

Older adults with disorders in mastication and swallowing are often fed soft foods, such as jelly or puree, rather than solid foods. Compensatory strategies for oral processing of food, such as squeezing the food with the tongue and palate, have been observed in patients with reduced masticatory function due to tooth loss (Ohta et al., 2017). Additionally, even healthy adults eating semi-solid foods can be observed to process the food by squeezing it with their tongue (Arai et al., 1992). In previous studies using electromyography (EMG), the activity of the suprahyoid muscles was observed when squeezing jelly with the tongue and elevating the tongue to the palate (Ishihara et al., 2011, 2013) and the activity of the suprahyoid muscles changed depending on the physical properties of the jelly.

A Lissajous figure expresses vector-synthesis on the Brownian *X*-axis and *Y*-axis. This method was able to describe the dynamic aspect of the muscle activity of two different muscles better than the level of myoelectric potential, and has been applied to express the sequential relationship between the two different muscles, for example the masseter and temporal muscles or bilateral muscles (Ferrario and Sforza, 1996; Ferrario et al., 1999, 2014). Moreover, the phase difference was obtained from Lissajous figure. We hypothesized that the Lissajous analysis should be able to clarify the differences in the sequential state of activity of the masseter and suprahyoid muscles caused by different feeding behaviors. This study aimed to test the hypothesis that there is a difference in sequential coordination between the masseter and suprahyoid muscles, and that feeding behaviors such as chewing and squeezing can be identified using EMG.

## Materials and Methods

### Subjects

The subjects were 17 healthy adult males (average age = 30.8 ± 4.2 years). We recruited them from university staff and students as volunteers after we presented the details of the experiment. All subjects provided written consent after receiving full written and verbal explanations of the purpose and details of the experiment. Exclusion criteria included any history of eating or swallowing difficulties, dysphagia, neurological disorders, dental pain, periodontal problems, or temporomandibular joint syndromes. Regarding the exclusion criteria, we did an oral interview about their medical history and confirmed that they had no subjective symptoms nor abnormal findings. We set the target number at 20 subjects because this study is an observational study, and there is a risk of exposure due to VF. However, only 17 eligible volunteers who applied for recruitment, therefore we adopted the largest possible number of subjects. This experiment was approved by the ethics committee of the Faculty of Dentistry of Niigata University (28-R2-4-14).

### Test Samples

As test samples, gels were prepared using a mixture of KELCOGEL^TM^ (low-acylated gellan gum) and KELCOGEL LT-100^TM^ (high-acylated gellan gum) (both from San-Ei Gen F.F.I., Inc., Osaka, Japan) (Ishihara et al., 2014; Table 1). Low-acylated gellan gum forms less deformable and more brittle gels than high-acylated gellan gum, and diverse textures can be obtained through blending of the two gums (Sworn, 2000). To mask the subtle flavor from these polysaccharides, which may have affected the results of sensory evaluation, sucrose was added at 10% (w/w) to all gel samples.

**TABLE 1:** Physical properties of samples.

Sample	Breaking load (N)	Breaking strain (%)
A10	9.71 ± 0.13	43.31 ± 0.34
A30	28.70 ± 1.00	46.16 ± 1.08
C10	9.73 ± 0.94	74.34 ± 1.67
C30	29.40 ± 0.99	78.71 ± 1.19

The mechanical characteristics of the gellan gels were measured using a TA. XTplus texture analyzer (Stable Micro Systems, Surrey, United Kingdom) through instrumental compression of the gel samples. We set the breaking load and strain of each gel samples. Breaking load and breaking strain were determined from the first peak (breaking point) of the compression curve as shown in Supplementary Figure 1. Breaking strain was calculated as the ratio of the deformation at breaking point to the initial height of gel. Breaking load and strain were measured by compressing these gels on a hard metal stage using a cylindrical aluminum plate 50 mm in diameter at a crosshead speed of 10 mm/s at 20°C. The gel sample was 20 mm in diameter and 10 mm in height. Compression rate was 90%. The average of the five samples was taken as the physical properties of each sample.

In this study, four kinds of prepared gel samples were used (A10, A30, C10, and C30). The breaking load was set to 30 N for A30 and C30, and 10 N for A10 and C10. The breaking strain was set to 45% for A10 and A30, and 75% for C10 and C30 (Table 1). In other words, A30 and C30 were formulated to have the same breaking load, and A10 and A30 to have the same breaking strain. The mechanical properties of the four test samples used in this experiment were as follows: A10 was soft and brittle, A30 was hard and brittle, C10 was soft and elastic, and C30 was hard and elastic. These gels were contrasted for videofluorography (VF) by 8.2 w/v% iopamiron 370^®^ (Bracco Imaging, Milan, Italy) and the taste was corrected with granulated sucrose. Additionally, subjects were provided with the test samples with all the conditions coordinated, such as the form (semi-spherical), size, color, and smell, so that each test sample could not be distinguished from other test samples except for the oral sensation when eating.

### Measurement Device

The EMG activity of the right masseter and supra-hyoid muscles was recorded during using a wired bipolar surface EMG system (NT-212u, Nihon Kohden, Tokyo, Japan). The two couples of electrodes were attached non-invasively to the skin surface over the part of corresponding to the masseter and supra-hyoid muscles at an inter-electrode distance of 20 mm so as not to restrict the subjects’ movements, while a ground electrode was applied to the right ear. The same operator performed the work of attaching the electrodes so that there was no variation among the subjects. After placing the electrodes, each subject waited for a certain period until the potential stabilized. The subjects were asked to perform occlusal movement, tongue elevation, and swallowing, and we confirmed that the EMG was recorded normally. All muscle activity was recorded from the intake of the test food to the initial swallowing at a sampling frequency of 1000 Hz. All signals were amplified with an amplifier (AB611-J, Nihon Kohden, Tokyo, Japan) and recorded on the computer through an analog to digital converter (Power Lab ML880, AD Instruments, Bella Vista, Australia). The EMG signals were then filtered (30–1000 Hz). The recorded EMG signals were analyzed after full-wave rectification waveform processing. To assess the feeding behavior during free ingestion, videofluorography (VF) was recorded at 30 frames/s (ARCADIS Avantic Gen2, Siemens, Germany) from the sagittal plane, and recorded on the computer through Power Lab ML880 simultaneously.

### Data Collection and Feeding Behavior

A semi-spherical gel sample was put on each subject’s tongue by the assistant in the same manner. The subjects sat on the examination chair and the headrest position was adjusted so that the subject’s Frankfurt plane was parallel to the floor.

The subjects were instructed to consume the test products in one of three ways: squeezing, chewing, or eating freely. For squeezing, the subject was instructed “Don’t chew but squeeze with your tongue.” The instruction for chewing was “chew with your teeth,” and the instruction for eating freely was simply “eat freely,” with no specific instructions. Squeezing with the tongue and chewing were performed twice for each sample (8 times in total), and eating freely was performed once for each sample (4 times in total). For each method, the subject consumed the sample until swallowing, with no limit on the amount and time of chewing or squeezing, and the order of implementation was randomized using online random number generator (Research Randomizer)^1^. The EMG activity was recorded when the test sample was ingested, and the VF was recorded from the sagittal plane at the same time.

### Data Analysis

After recording the entire EMG activity of the masseter and suprahyoid muscles during chewing and squeezing with the tongue, only the first stroke of chewing or the first squeezing with the tongue were extracted as the target and analyzed. The first stroke of chewing or squeezing with the tongue was defined as the lowest value including one peak value to the next lowest value.

The Lissajous figures (scatter charts) were created by plotting the values of the EMG activity of the suprahyoid muscles on the *X*-axis and the values of the masseter muscle on the *Y*-axis. Then, a regression line was made on these Lissajous figures to determine the gradients (Supplementary Figure 2). Then the gradient of regression line of the EMG activity of the masseter and supra-hyoid muscles during the first stroke was calculated and compared between squeezing with the tongue and chewing using the Mann–Whitney *U*-test. Additionally, using the gradient values of regression line of squeezing and chewing data, the receiver operating characteristic (ROC) curve for distinguishing feeding behaviors was drawn. The optimum cutoff value for these behaviors was established using the receiver operating characteristic (ROC) curve.

The free intake was analyzed in the same manner. The free intake feeding behaviors were determined according to the cutoff value we obtained; in other words, the same analysis was performed during free intake, and the gradient of the regression line of the Lissajous figure was calculated and the feeding behaviors of squeezing with the tongue and chewing were compared. The consistency of the results was determined by observing the VF images. For the VF images, we focused on the gel’s first movement after it had been placed on the tongue. Chewing was determined to start when the gel was moved by the tongue onto the dental arch at the start of consumption and squeezing with the tongue was determined to start when it was raised and squeezed between the palate and the tongue.

The analysis software used was SPSS Statistics (ver. 25 IBM Japan, Tokyo, Japan) with the significance defined at *P* < 0.05.

## Results

**FIGURE 1:**
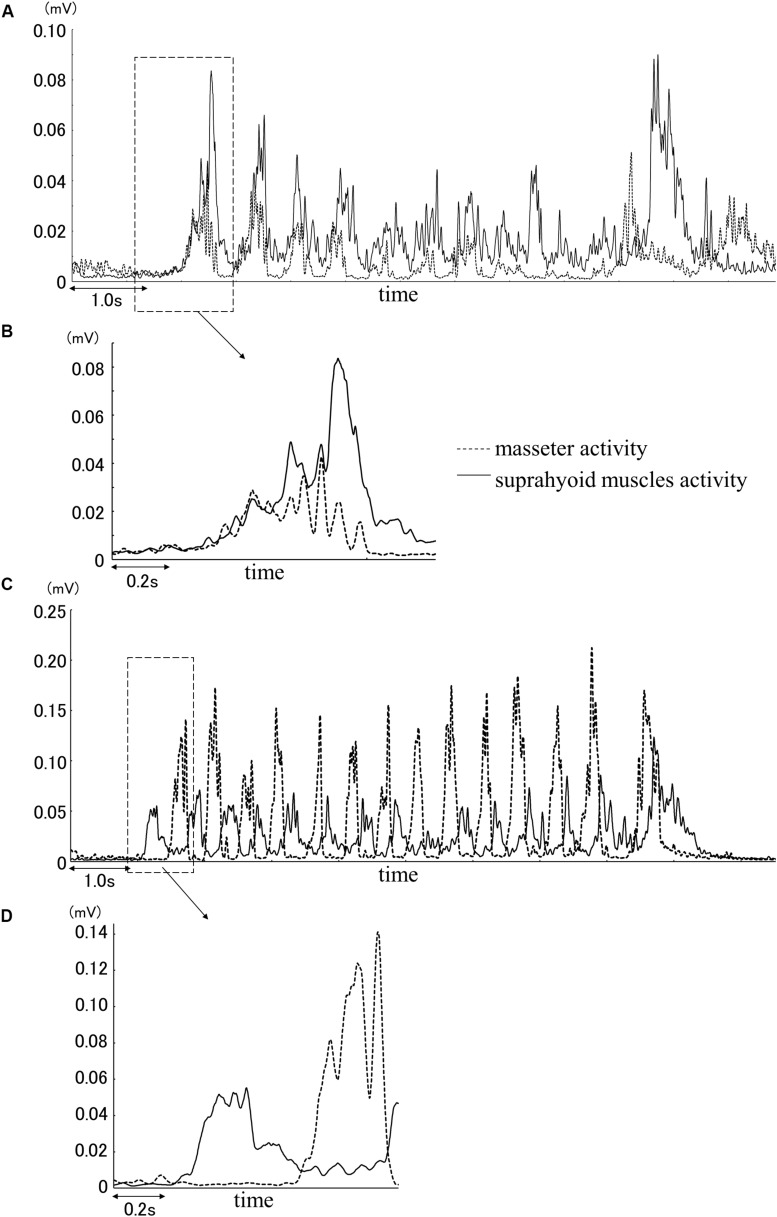
Example of muscle activity during squeezing **(A)** and the first cycle from the muscle activity data of the masseter and suprahyoid muscles **(B)**. Example of muscle activity during chewing **(C)** and the first cycle from the muscle activity data of the masseter and suprahyoid muscles **(D)**.

The masseter and supra-hyoid muscles were active almost simultaneously during squeezing (Figures 1A,B), whereas during chewing, the supra-hyoid and masseter muscles were active in turn during mastication (Figures 1C,D).

**FIGURE 2:**
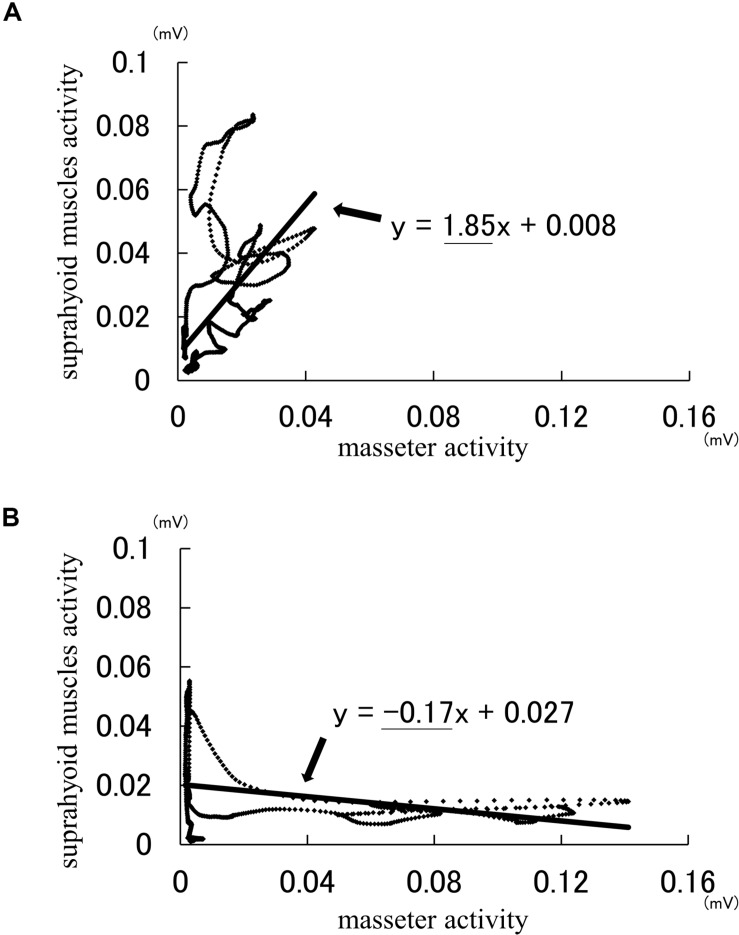
Example of the Lissajous figures for squeezing **(A)** and chewing **(B)**. The arrow indicates the regression line. The formulas in figures are the regression formula of regression line. The coefficient of x (underlined part) is the gradient value of regression line.

**FIGURE 3:**
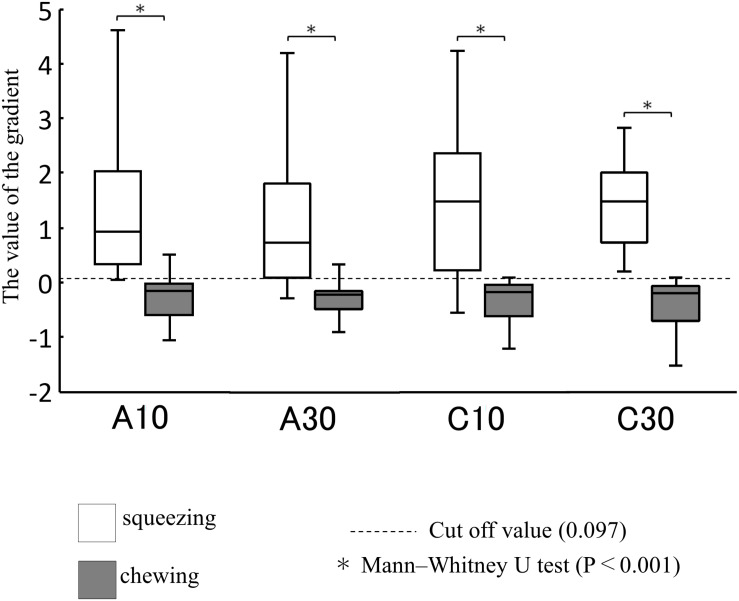
Comparison of gradient for each sample between chewing and squeezing (*n* = 136). The figure shows the median and interquartile range for each sample and the horizontal dashed line shows the cutoff value of 0.097.

**FIGURE 4:**
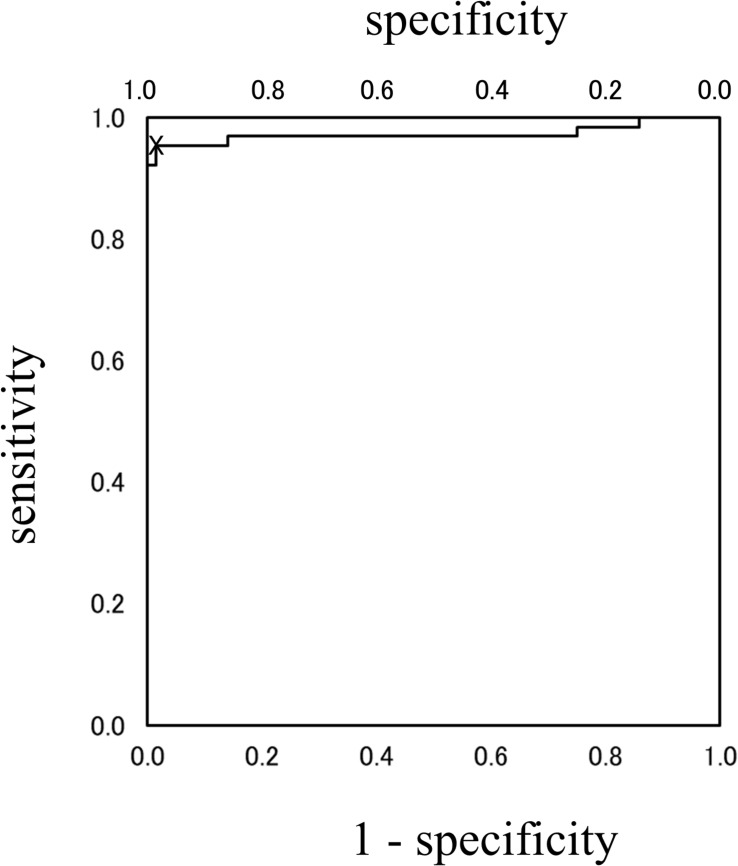
ROC-curve. The cutoff value of the gradient for differentiating feeding behaviors was 0.097. At point X, the best sensitivity was 95.3% and the specificity was 98.4%.

Analysis using the Lissajous figures of muscle activity showed that the gradient of the regression line was negative for chewing (Figure 2A), but positive for squeezing (Figure 2B). This tendency was common in all gel samples, and the value of inclination for squeezing was significantly greater than that for chewing (Figure 3). Examination of the cutoff value for the gradient of the regression line estimating the differences in feeding behavior using the ROC curve indicated that when the gradient was 0.097, the best sensitivity was 95.3% and the specificity was 98.4% (Figure 4).

Based on the cutoff value obtained, 68 free intakes were estimated and compared with the results of the VF determination (Table 2). The accuracy was 86.8%, the sensitivity was 91.1%, and the specificity was 66.7%. Additionally, only 2 out of 17 subjects performed squeezing with the tongue in more than half of the trials during free eating, and most subjects chewed when they ingested freely with all four test samples.

**TABLE 2:** Differentiation of feeding behavior based on videofluorography (VF) and the cutoff value.

		Determined by cutoff value (0.097)	
		
		Chewing	Squeezing
Determined by VF	Chewing	51	5
	Squeezing	4	8

## Discussion

In this study, we were able to distinguish two types of feeding behaviors, squeezing with the tongue and chewing, by using a new method of analyzing Lissajous figures of the phase differences of the masseter and suprahyoid muscles. This method was a way to allow distinguishing between two gradients of regression lines, one for chewing and the other for squeezing. The regression line was obtained from the Lissajous figure, i.e., putting an EMG of supra hyoid muscles on the *X*-axis and the EMG of masseter on the *Y*-axis and determining the regression lines (Supplementary Figure 3). The results of this study show the feasibility of using a non-invasive method for differentiating feeding behaviors. This method could be used at facilities such as nursing homes where there is no professional who can evaluate chewing and swallowing function, and where determination of the feeding behavior relies on self-reporting by the patient. Objective and quantitative assessment of feeding behavior could be helpful in determining the most appropriate food for patients, thus reducing the risk of suffocation during meals in disabled older adults.

### Differences in Muscle Activity Between Chewing and Squeezing

During chewing, the masseter muscle was active during jaw-closing and the suprahyoid muscle was active during jaw-opening. As a result, masseter muscle activity was observed after suprahyoid muscle activity, and the Lissajous figure suggested the phase difference in the activity of the masseter and sura-hyoid muscles. In contrast, when squeezing with tongue, not only the supra-hyoid muscles were working, but also the masseters were active asynchronously. Therefore, it can be deduced that the tongue was raised while the mouth was closed and the food was being compressed during squeezing. In most subjects, the EMG data for the masseter tended to be smaller during squeezing with the tongue than when chewing (data not shown). During chewing, the masseter muscle contracts until the upper and lower teeth are firmly engaged, but when squeezing with the tongue, there is no need to bite deeply as is required during chewing. Thus, it can be deduced that the main role of the masseter muscle during squeezing with the tongue is to work in cooperation with the supra-hyoid muscle to close the mouth and fix the lower jaw in place.

In this study, we measured the masseter and suprahyoid muscles of the right side only. The jaw movement represented by mastication is an activity resulting from the cooperation of the paired left and right temporomandibular joints and the masticatory muscles. It is known that there is a slight left–right difference in that the muscle activity on the mastication side occurs first. In this study, the main purpose was to analyze the difference in cooperative EMG activity between different muscles related to feeding behavior. Therefore, we only analyzed the masseter and suprahyoid muscles on the right side, to determine their different roles in chewing and squeezing with the tongue.

### Analysis of Free Intake

In the free ingestion task of the present study, only 2 out of 17 subjects performed squeezing with the tongue for more than half of the four types of samples, and most subjects performed chewing with most of the samples. All the test samples were classified as “food for people who have problems with chewing” according to the Smile Care Food classification, which is one of the standards for the classification of nursing home foods established by the Ministry of Agriculture, Forestry and Fisheries. A30 was a food that can be easily chewed (Smile Care Food 5), A10 and C30 were foods that can be crushed by the gums (Smile Care Food 4), and C10 was a food that can be crushed by the tongue (Smile Care Food 3). These four kinds of test foods were selected because we expected that the feeding behavior would change depending on the physical properties of the food. However, in reality, many subjects chewed all the test foods when they were ingested freely. These outcomes suggest that “food that can be eaten by squeezing with the tongue” was not necessarily “food that you want to eat by squeezing with the tongue” for all subjects. The following factors were considered to have greatly affected the results: (1) all subjects were healthy dentulous adults; (2) there were no extreme differences in the physical properties of the four test samples; and (3) gel sample had a slight bitterness due to contrast agents. Furthermore, these food were developed for elderly patients of the patients with dysphagia. Therefore, verification for the elderly is needed in the future.

It is speculated that these results might be due to the fact that the subjects were adults with healthy teeth and that there were no extreme differences in the physical properties of the four test samples used in this study. Past studies on mastication suggested that factors that affect masticatory movement include food size, food hardness, and the condition of the subject (Mizumori et al., 1985). In this study, all subjects were fed gel samples of the same size (volume) by the operator. According to a study by Arai et al. (1992) which investigated the effect of food shape on mastication, the most important factor for differentiating feeding behaviors when ingesting semi-solid foods such as agar gels was the size, rather than the physical properties of the gel. Additionally, when the test food was 15 × 15 × 15 mm or less, all subjects ingested the sample by squeezing with the tongue and their hard palate, but when the test food was 20 × 20 × 15 mm or more, the main ingestion pattern changed from squeezing to chewing. They also reported that the food size threshold for changing feeding behavior was 20 × 20 × 15 mm (Arai et al., 1992; Fueki et al., 2008). The test samples we used in this study were equivalent to one teaspoon (5 ml), with a diameter of 25 mm and height of 15 mm. This is slightly larger than the threshold of 20 × 20 × 15 mm, as suggested by Arai et al. (1992) which may have increased the percentage of subjects who performed chewing during free eating.

Bellisle et al. found that the chewing time became shorter when subjects ate something delicious and enjoyed rolling the food on the tongue (Bellisle et al., 2000; Johansson et al., 2013). It is thought that the preferences and experience of each subject are key determinants in choosing whether to chew or squeeze a food with the tongue. However, although the test samples used in this study were flavored with granulated sugar, the characteristic bitter flavor of the iopamiron370^®^ remained, and the food was never thought to be delicious. The effect of the bitter flavor of the test sample could be the reason why fewer subjects ingested by squeezing with the tongue.

### Limitations and Clinical Significance

In this study, the number of subjects and experimental tasks was limited to minimize the radiation exposure. Because we analyzed only the first cycle of each feeding behavior in this study, we were able to discern the trend of the beginning of each feeding behavior. In this report we focused on developing and testing the methodology for using Lissajous figures to distinguish tongue squeeze and chew behaviors. We think that it is important to be able to objectively quantify the feeding behavior. To identify changes in the characteristics of each feeding behavior over time and to evaluate the strategy of oral processing of food, we should conduct an analysis using Lissajous figures with the EMG data from the masseter and suprahyoid muscles from the start of feeding to swallowing. Further analysis of these data might provide important insights in the future.

It is reported that cognitive function, occlusal support including dentures, and oral function are important for preventing choking (Ariyoshi et al., 2013). Some elderly people, even if they retain a complete dentition, eat with tongue squeezing instead of chewing. Preventing choking or aspiration and providing safe meals involving both behaviors are important goals for monitoring feeding by elderly patients (Jayatilake et al., 2015). In order to assess what kinds of foods might best be eaten by specific individuals, reliable discrimination of chewing and tongue squeezing is important. However, reliable discrimination by experienced observers might be difficult and error prone in a busy clinical environment. The methods proposed in this study for rapid and reliable assessment of food oral processing behavior would ideally be validated in elderly people, but it is difficult to measure VF and EMG synchronously in elderly people. Future research should compare the results of our experiment with those of a similar experiment with older adults with missing teeth. It would be expected that the ratio of chewing in free eating would be different from that of young subjects with healthy teeth.

A lot of work is needed to develop a clinical application of Lissajous figures to determine feeding behavior. However, a small electromyograph has been released recently (Yamaguchi et al., 2018) and the data analysis method used in this report was not very complicated. It is important to be able to evaluate feeding behavior by objective numerical values, regardless of the caregiver’s experience, in the care of the elderly. We consider this report to be the first step. Moreover, this method might also be applied in food engineering for designing food for the elderly requiring care.

## Conclusion

By analyzing the muscle activity of the masseter and suprahyoid muscles using Lissajous figures, the cutoff value 0.097 was determined for the eating pattern of tongue crushing and mastication. These results suggest that this method may be a new means for non-invasive assessment of solid food consumption.

## Data Availability Statement

The datasets generated for this study are available on request to the corresponding author.

## Ethics Statement

The studies involving human participants were reviewed and approved by the Ethics Committee of the Faculty of Dentistry of Niigata University. The patients/participants provided their written informed consent to participate in this study.

## Author Contributions

KH designed the study. FU, KM, JO, and KH collected the data. FU and JO analyzed the data. FU and KH drafted the manuscript. TO edited the manuscript.

## Conflict of Interest

The authors declare that the research was conducted in the absence of any commercial or financial relationships that could be construed as a potential conflict of interest.

## Footnotes

1 https://www.randomizer.org/
