# Supplementary figures and images for: Differentiation of Feeding Behaviors Based on Masseter and Supra-Hyoid Muscle Activity
Source: Front Physiol. 2020 Jun 12;11:618. doi: 10.3389/fphys.2020.00618 (PMC7303331; doi:10.3389/fphys.2020.00618)

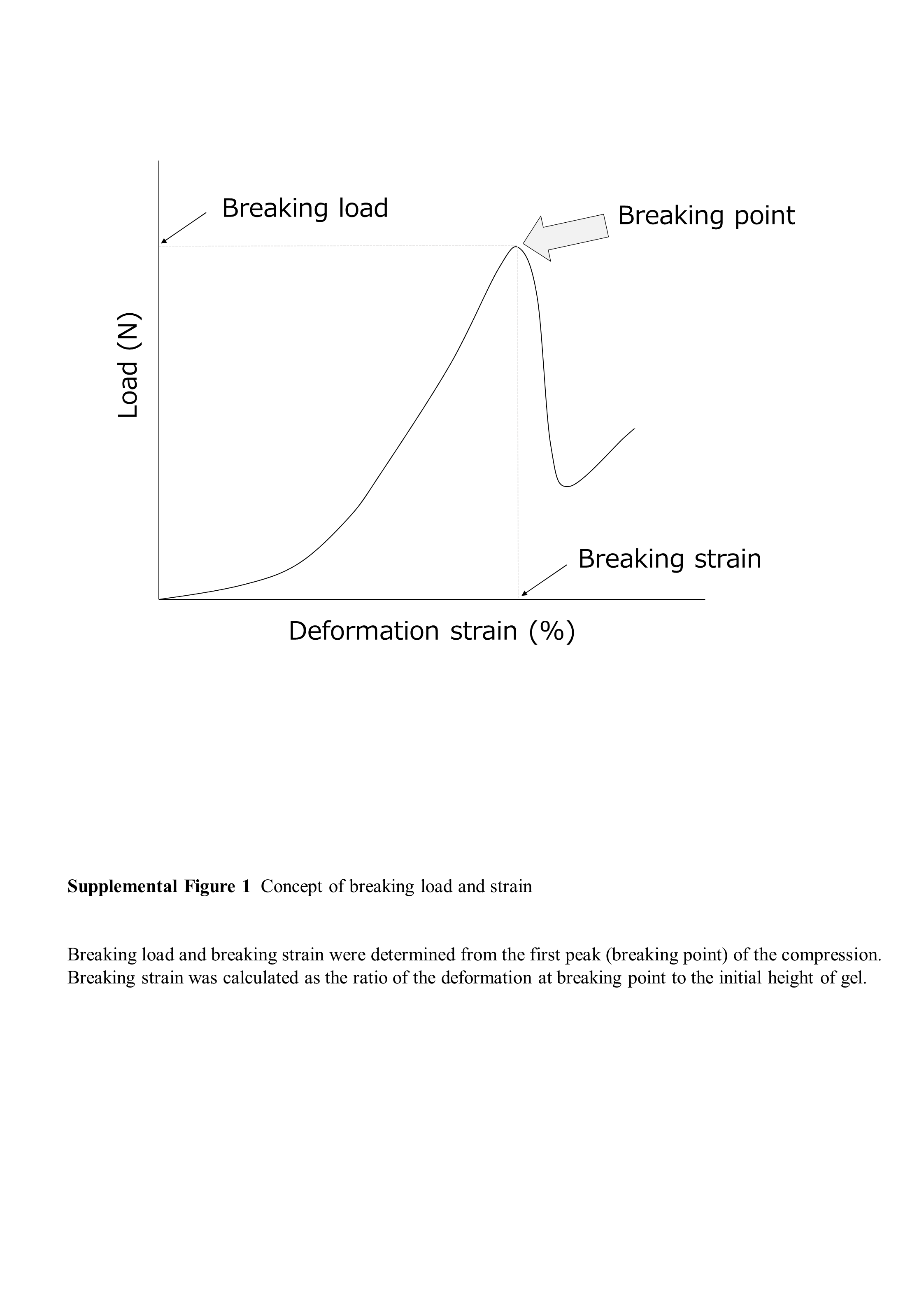

Supplement: Supplementary file 1 [file Image_1.TIF]

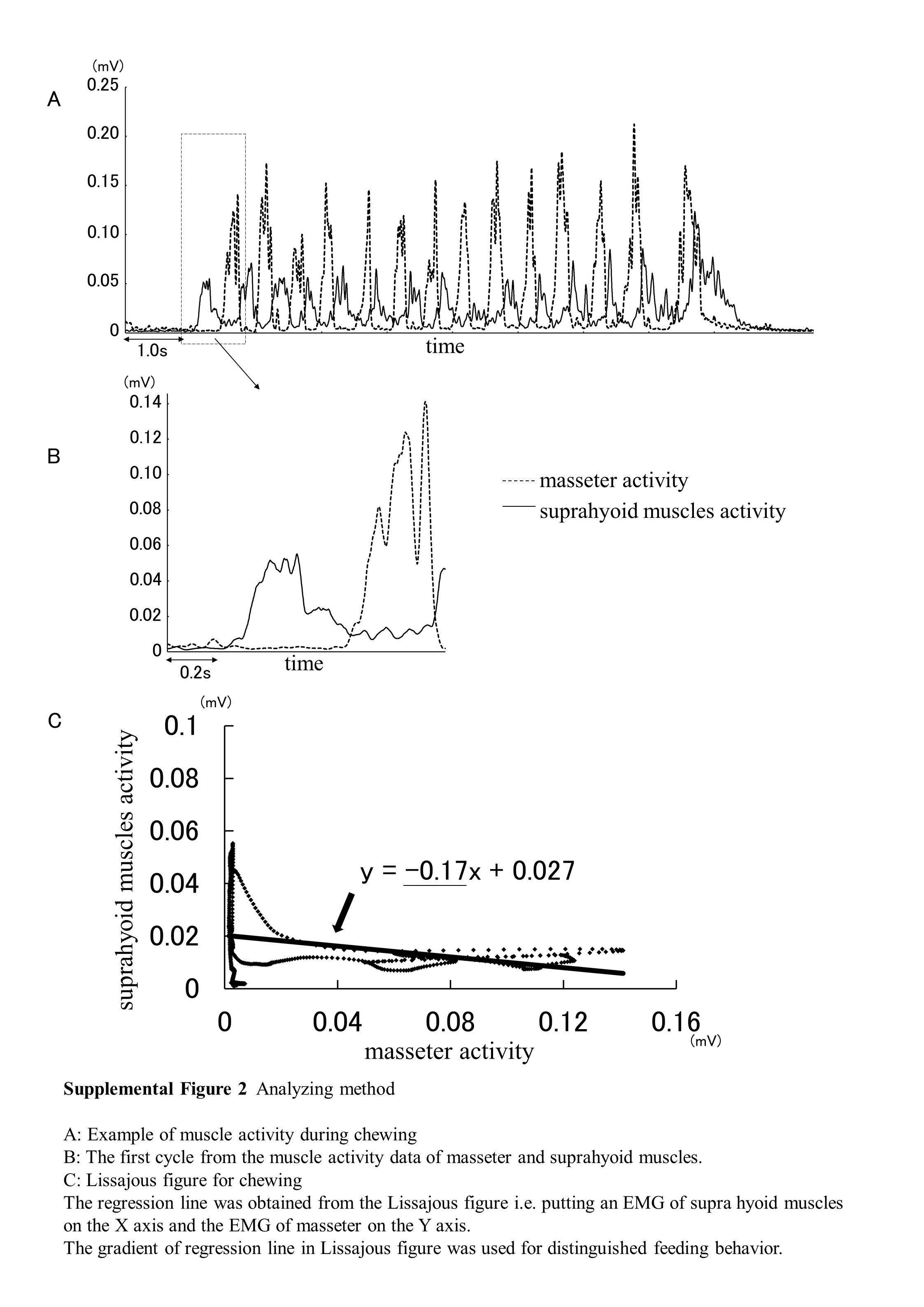

Supplement: Supplementary file 2 [file Image_2.TIF]

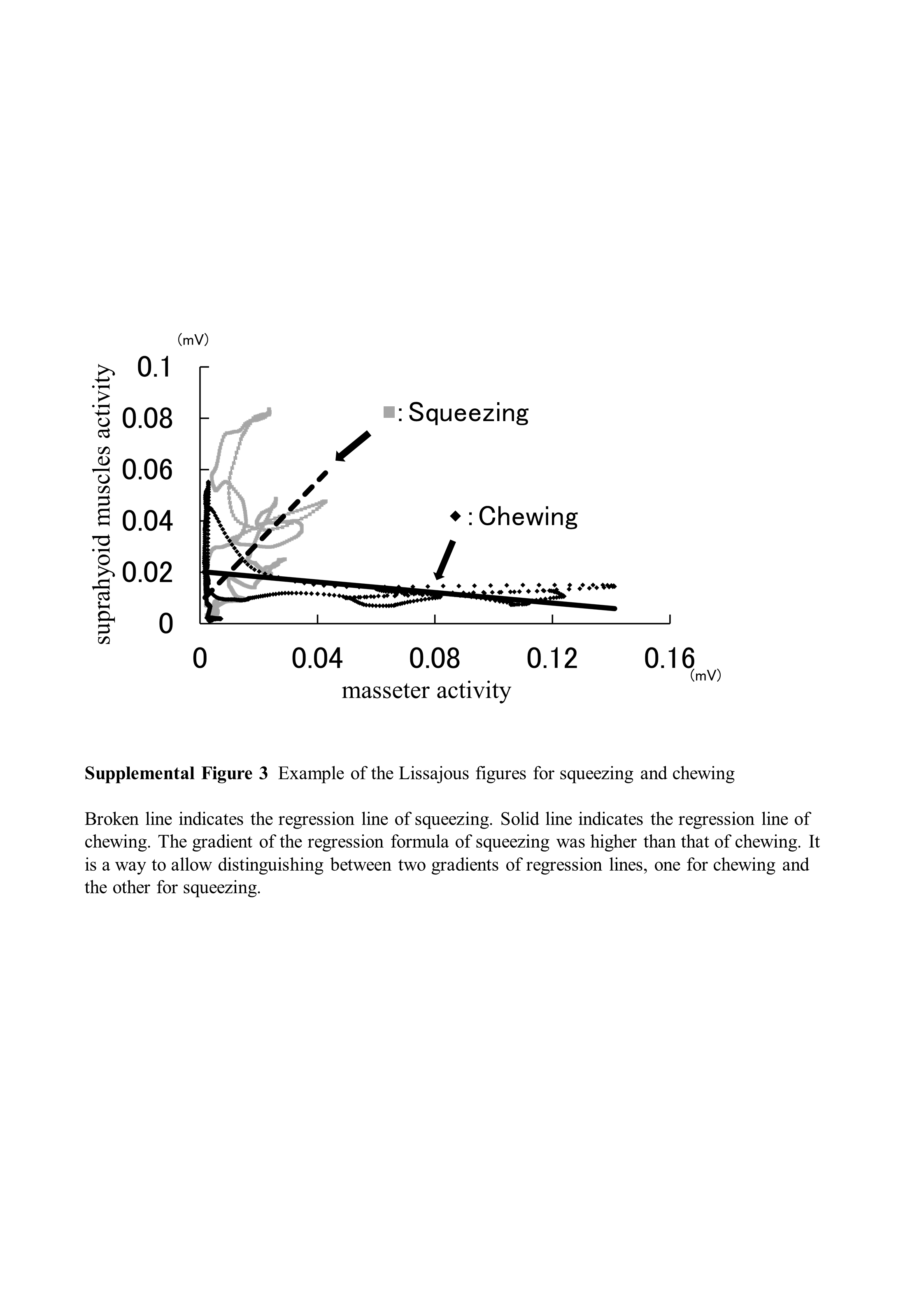

Supplement: Supplementary file 3 [file Image_3.TIF]
